# Do coastal salt mudflats (sabkhas) contribute to the blue carbon sequestration?

**DOI:** 10.1007/s10533-024-01204-5

**Published:** 2025-01-15

**Authors:** Hadil Elsayed, Zulfa Ali Al Disi, Khaled Naja, Ivan Strakhov, Scott O. C. Mundle, Hamad Al Saad Al-Kuwari, Fadhil Sadooni, Zach Diloreto, Jassim Abdulla A. Al-Khayat, Maria Dittrich

**Affiliations:** 1 Environmental Science Center, Qatar University, P.O. Box 2713, Doha, Qatar; 2 Biogeochemistry Group, Department of Physical and Environmental Sciences, University of Toronto Scarborough, 1065 Military Trail, Toronto, ON M1C 1A1 Canada; 3 University of Windsor, 401 Sunset Ave, Windsor, Ontario N9B 3P4O Canada

**Keywords:** Sabkha, Tidal mudflats, Carbonates, Carbon sequestration, Carbon stock, Coastal ecosystem

## Abstract

Blue carbon represents the organic carbon retained in marine coastal ecosystems. *Sabkhas* (an Arabic for “mudflats”), formed in tidal environments under arid conditions, have been proposed to be capable of carbon sequestrating. Despite the growing understanding of the critical role of blue carbon ecosystems, there is a current dispute about whether sabkhas around the Persian Gulf can contribute to carbon retention as a blue carbon ecosystem. The arguments often lack data on a critical contributor, inorganic carbon in the form of carbonates, which can drive the net carbon exchange with the atmosphere. In this study we inventory organic and inorganic carbon retention capacity in two contrasting sabkhas of the Qatar Peninsula: carbonaceous Dohat Faishakh and siliciclastic Khor Al-Adaid. Despite the differences in organic carbon stock between the two sabkhas, the Dohat Faishakh sabkha has higher (37.17 ± 0.81 Mg C_org_ ha^−1^) than it is in the Khor Al-Adaid sabkha (13.75 ± 0.38 Mg C_org_ ha^−1^) for 0. 44 m sediment depth, the organic carbon retained in sabkhas is similar to those reported for mangroves and salt marshes. Notably, calculated CO_2_ net sequestration indicated that both sabkhas evade CO_2_ into the atmosphere. Thus, carbonate formation negated organic carbon accumulation in carbonaceous sabkha. Consequently, for proper evaluation of sabkhas as a blue carbon ecosystem, an inorganic carbon analysis, especially of carbonate formation, is inevitable. Considering only organic carbon stock may ay overestimate carbon sequestration capacity.

**Supplementary Information:**

The online version contains supplementary material available at 10.1007/s10533-024-01204-5.

## Introduction

Blue carbon is the carbon captured and stored in coastal sediments (Macreadie et al. 2019). Blue carbon ecosystems (BCEs) have been attributed to coastal wetland ecosystems with manageable and atmospherically significant carbon stocks and fluxes—such as mangroves, salt marshes, and seagrass—and contribute to 50% of the global carbon burial (Duarte et al. 2013; Windham-Myers et al. 2018). BCEs have been found to contribute to the regulation of Earth’s climate, and it has been proposed that preserving and enhancing these ecosystems could help in the mitigation of, and adaptation to, climate change (Hilmi et al. 2021). Studies on vegetated coastal habitats such as mangroves, seagrass, and salt marshes have confirmed their capacity to sequester and store significant amounts of organic carbon: 255, 140, and 162 Mg C ha^−1^, respectively (Cusack et al. 2018; Lee et al. 2021). Coastal ecosystems can sequester carbon at rates that are up to 30 to 50 times greater than those of terrestrial forest ecosystems due to their anaerobic conditions, which result in slower decomposition rates that allow for more accumulation of organic carbon in sediments over time (Mcleod et al. 2011; Taillardat et al. 2018), thus giving them an important role in sequestration strategies and climate change mitigation (Moritsch et al. 2021).

While extensive studies on blue carbon have been conducted in mangrove forests and coastal sea basins, less is known about organic carbon within intertidal zones (Table 1). One type of intertidal ecosystem that can be designated as a possible BCE are sabkha environments. Sabkhas are coastal hypersaline mudflats that formed over time periods when the rate of evaporation exceeded the rate of rainfall (Whitaker et al. 2014), and they represent a diagenetic modification of sediments of marine origin (Moore and Wade 2013). Coastal sabkhas are often characterized by the presence of living microbial mats, which result in pronounced biogeochemical cycling of carbon, nitrogen, phosphorus, and trace elements (DiLoreto et al. 2019; Dupraz et al. 2009; Nutman et al. 2016). Microbial mats were suggested to play a potential role in blue carbon dynamics (Schile et al. 2017; Al disi et al. 2023). The microbial mats in Qatar’s sabkhas have been of interest with respect to carbonate formation and have also been considered an analogue for ancient sedimentary sequences (Wells 1962; Illing et al. 1965; Paulo and Dittrich 2013; Brauchli et al 2016; DiLoreto et al. 2019). As intertidal environments, sabkhas with low vegetation cover play an unknown role in blue carbon sequestration (Schile et al. 2017; Lovelock and Duarte 2019); in particular, the sabkhas located in Qatar are understudied with respect to carbon retention (Neue et al. 1997; Amundson 2001; Eid et al. 2022). Various abiotic and biotic factors including, the anthropogenic pressure impact the soil organic carbon (SOC) stocks.

**Table 1:** Summary of organic carbon stock data studied in different ecosystems, along the shores of the Arabian Gulf and the Red Sea

Coastal ecosystem	Country	C_org_ stock, Mg C ha^−1^	References
Seagrass	UAE	2.2–109.3	Schile et al. (2017)
Seagrass	Saudi Arabia	55–127	Cusak et al. (2018)
Microbial mats	UAE	18.6–242.4	Schile et al. (2017)
Mature mangrove	UAE	77.4–515.5	Schile et al. (2017)
Planted mangrove	UAE	51.3–182.3	Schile et al. (2017)
Mangrove	Saudi Arabia	51–113	Cusak et al. (2018)
Mangrove	Qatar	6.23–33.74	Chatting et al. (2020)
Salt marsh	UAE	31.4–205	Schile et al. (2017)
Salt marsh	Saudi Arabia	58–113	Cusak et al. (2018)
Coastal sabkha	UAE	51–120	Schile et al. (2017)
Coastal sabkha	Qatar	67.8–109	Al disi et al. (2023)
Coastal sabkha	Qatar	31–84	This study

The enhancement of construction activities along the shorelines in Qatar and other Arabian Gulf countries led to the drastic degradation of sabkha ecosystems. Many sabkhas coastal ecosystems have been destroyed through the years due to urban development. While more experimental data on the rates of carbon accumulation are required to estimate the role of sabkhas in climate change mitigation, it is imperative that sabkha environments are preserved and can be studied.

Sabkhas are slow-evolving, slow-establishing ecosystems, and their restoration requires long-term strategic planning and knowledge of system functioning. Thus, the present study on Qatari sabkhas can be applied to similar coastal ecosystems, and can have global implications for the carbon balance and for supporting the conservation of vunerable ecosystems and the mitigation of ecological degradation under changing climatic conditions.

While significant progress has been made in understanding blue carbon ecosystems, less attention is given to intertidal zones in the Arabian Gulf. Recent studies on coastal sabkhas along the southern Red Sea coast of Saudi Arabia estimated the SOC stocks to be from 36 Mg C/ha to 194 Mg C/ha based on 50 cm depth, depending on vegetation cover (Eid et al. 2022, 2023; Al disi et al. 2023). The relatively low SOC may be a result of environmental conditions, e.g., high salinity, poor soil texture. The sabkhas of Qatar have been a subject of research focusing on their productive microbial mats, carbonate formation, and biogeochemical cycles (Wells 1962; Illing et al. 1965; Paulo and Dittrich 2013; Brauchli et al. 2016; Al disi et al. 2019; DiLoreto et al. 2019).

Thus, in this study we compare SOC stock and CO_2_ sequestration and evasion from the sabkhas located in the contrasting geological backgrounds, with microbial mats serving as the primary source of organic matter. We hypothesize that organic matter retention depends not only on the vegetation cover but also on the geological background. With the present study, we want to draw attention to the ecological role of sabkhas in terms of carbon sequestration, highlighting the need for sabkha conservation and protection. The specific objectives of this study are (i) to examine the sabkhas’ potential for carbon storage considering organic and inorganic carbon, (ii) to compare their carbon retention to those of other coastal ecosystems, and (iii) to shed light on the role of geological and geochemical conditions on carbon retention in evaporitic coastal flatlands.

## Materials and methods

### Site description

**Fig. 1:**
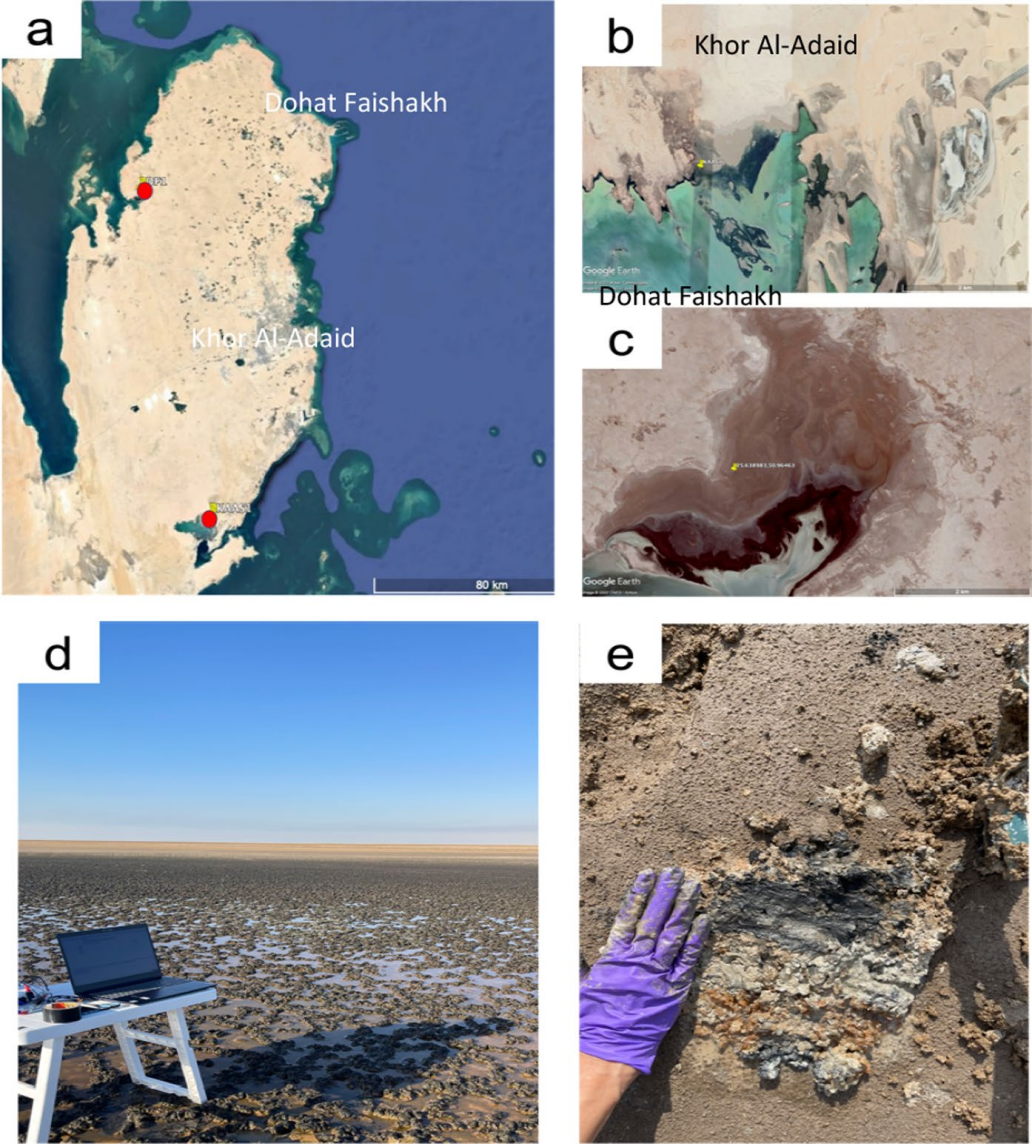
**a** Map of Qatar; **b** satellite image of the Khor Al-Adaid sabkha and **c** satellite image of the Dohat Faishakh sabkha; **d** photograph of the Khor Al-Adaid sabkha; **e** photograph of the surface of the Dohat Faishakh sabkha

We investigated two sabkhas: the Khor Al-Adaid sabkha (51.326946, 24.645705) in October 2020 and the Dohat Faishakh sabkha (25.638983, 50.96463) in April 2021 (Fig. 1). The Khor Al-Adaid sabkha is in the southeast of Qatar, within a large tidal embayment constituted of two marginal inland lagoons (Fig. 1b, d). It is a typical hypersaline sabkha covered with microbial mats, surrounded by large sand dunes; the sediments of this sabkha are dominated by siliciclastic particles (Paulo and Dittrich 2013). The Dohat Faishakh sabkha is located on the northwest coast of the Qatar Peninsula (Fig. 1c, e). It is an evaporitic environment, and its sediments are dominated by gypsum and carbonate minerals that formed during the Holocene (Illing et al. 1965; Whitaker et al. 2014; Strohmenger and Jameson 2015; Brauchli et al. 2016;).

### Sabkha sampling: sediments and porewater

**Fig. 2:**
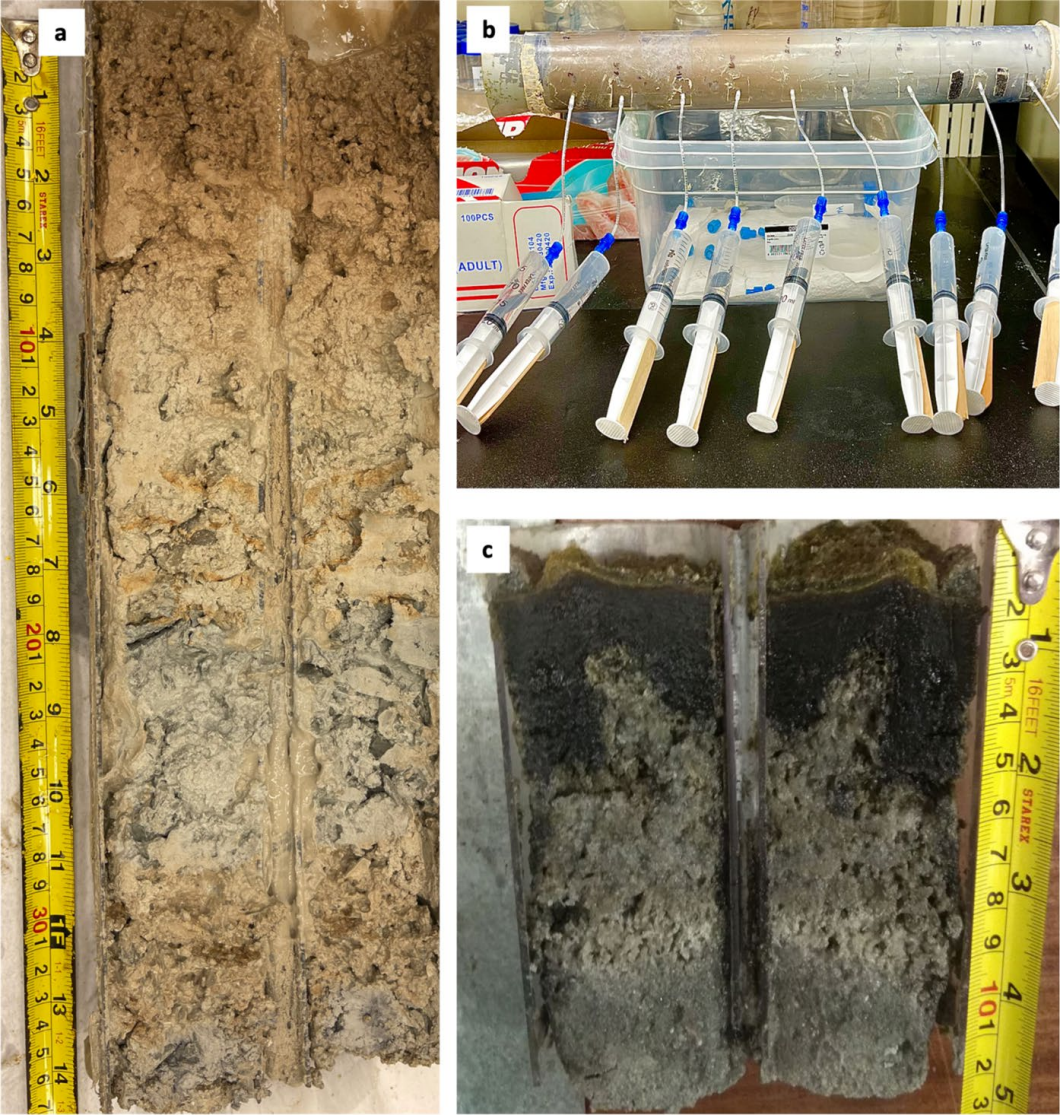
**a** Open core from the Khor Al-Adaid sabkha with different stratification. **b** Open core from the Dohat Faishakh sabkha with different stratification

Soil was sampled using a soil corer with a diameter of 6 cm and a length of 50 cm; at least two cores were collected from each site, one of the cores was equipped with holes along the length at 2 to 5 cm apart, depending on different stratification (Fig. 2). The soil corer was manually inserted downward 30 to 50 cm into the soil (Eid et al. 2022). Porewater was obtained using a Rhizon™ (Rhizosphere Research Products, Wageningen, the Netherlands), which naturally filters extracted porewaters at 0.2 μM Rhizons were deployed into pre-drilled holes at regular depth intervals along the sediment cores. The collected porewater was transferred to sterile 15 mL centrifuge tubes and preserved by adding 10 μL of saturated HgCl_2_ to every 10 mL until further analysis.

The cores were transported to the laboratory at Qatar University, Doha, within 3 h after sampling and were immediately processed. Sediment cores were sectioned into 2 to 5 cm layers, starting from the surface layer to 5 and 10 cm layers down to a depth of 44 cm. About 15 g from each sectioned layer was transferred to sterile tubes for solid matter characterization. Sediment samples were desalted by washing with distilled water, and then freeze-dried (Wang and Lin 2004). Five grams of freeze-dried sediments were manually ground using a mortar and pestle before the geochemical analysis was performed.

### Geochemical characteristics

#### Total carbon and total inorganic carbon content of sediments and porewater

The total carbon content of the sediment samples was analyzed using a CHNS Skalar Primacs™ SNC-100 TN/TC/IC analyzer. The sediment samples were ground to a particle size of approximately 0.05 mm, and 75 to 125 mg of the sediment were used for analysis. The samples were combusted with pure O_2_ at 1200°C to allow for the complete oxidation of carbon to CO_2_; the CO_2_ was then measured by infrared (IR) for total carbon. For measuring inorganic carbon, the samples were acidified using 10% phosphoric acid and the produced CO_2_ was detected through IR. Organic carbon was calculated by subtracting total carbon from inorganic carbon. Total organic carbon in the porewater was measured using an Analytik Jena 3100 N/C using EPA methods 415 and 9060A (EPA 1986). Results of TOC and TIC are reported in weight percent (wt%).

#### Mineralogical composition

The bulk mineralogical composition of the sediments was determined using X-ray Diffraction (XRD). Measurements that were performed on the PANalytical Empyrean diffractometer operating in the reflection mode with Cu (radiation (45 kV, 40 mA)) and diffracted-beam monochromator and a step size °2Theta of 0.0130; measurements were done at 25 °C. The analysis of XRD spectra was performed using Crystal Impact Match! software, Version 3.12 (https://www.crystalimpact.com/match/). The amounts of the minerals in each mixture were semi-quantitatively assessed using ICDD PDF database product as a reference database (Gates-Rector & Blanton 2019).

#### Bulk density, carbon stock, and theoretical CO_2_ net sequestration

Directly after arriving at the laboratory, the cores were sectioned and the bulk density of the sediment was estimated (Al-Shammary et al. 2018). At each depth, 5 mL volumetric cylinders were sub-sampled into each section of the sediment. The soil samples were dried in the oven at 105°C for 2 days; the weight of the sample was recorded before and after drying. The dry soil bulk density was estimated using the formula (1):

1 $$ \rho b = M_{{\text{s}}} /V_{{\text{s}}} , $$

where *ρb* is in Mg m^−3^, *Ms* is the weight of the dry soil sample in Mg, and *Vs* is the volume of the dry soil sample in m^3^.

C_org_ density (g C_org_ cm^−3^) was calculated for each sediment depth in each core by multiplying the sediment dry bulk density (g cm^−3^) by the C_org_ concentration (%). Sediment C_org_ stocks in each layer were calculated using the formula (2) below:

2 $$ {\text{Sediment }}C \left( {{\text{Mg ha}}^{ - 1} } \right) = {\text{Bulk density}} \left( {{\text{g cm}}^{{3}} } \right) \times {\text{Sediment depth}}\, \left( {{\text{cm}}} \right) \times \% C $$

Sediment C_org_ at a given depth was estimated as the summation of C_org_ stocks in all sediment layers. Then, sediment C_org_ stocks per unit area were extrapolated to a depth of 0.44 m to facilitate comparison with other studies.

Theoretical CO_2_ net sequestration was estimated using (Smith 2013; Howard et al. 2018), defined as the total calculated flux of CO_2_ from the atmosphere to coastal sediments, indicating that negative values of CO_2_ net sequestration show evasion of CO_2_ from seagrass soils to the atmosphere (Howard et al. 2018). We applied this Eq. (3) to sabkha sediments:

3 $$ C_{{{\text{org}}}} stored - \psi \times C_{{{\text{inorg}}}} = {\text{CO}}_{{2}} net\, sequestration, $$

where C_org_ stored is the C_org_ density in (mol mL soil),

ψ is the gas exchange: reaction ratio of CO and CaCO_3_ (dimensionless) proposed by Smith (2013),

C_inorg_ stored is the C_inorg_ density in (mol mL soil), and CO_2_ net sequestered is the amount CO_2_ sequestered in (mol mL soil).

#### Carbon dating

The carbonates samples collected from cores from Khor Al-Adaid at 35 and 40 cm were chosen for radiocarbon dating, which was done using accelerator mass spectrometry (AMS) at the BETA Analytic testing laboratory in Florida, USA.

#### Gas composition analysis

For gas composition analyses, the samples were collected in pre-evacuated 160 mL glass vials fixed with HgCl_2_ and sealed with blue butyl rubber stoppers after the method of Ward et al. (2004). Prior to collecting the sample, sample gas was allowed to flow for approximately 10 min to flush out the reservoir and ensure a representative sampling. Gas was then sampled from the sabkha with a syringe and then injected into the pre-evacuated vial. CO_2_ concentrations were analyzed using an Agilent 7890B gas chromatograph equipped with a thermal conductivity detector. Two depths have been chosen for CO_2_%, which are 5 cm and 22 cm, to compare between CO_2_ levels underneath surface sediments which is undergoing a lot of fast changes and deeper sediments.

#### Analysis of major and trace elements

For the analysis of major and trace elements (Ca, S, Be, Mg, Al, P, K, V, Cr, Mn, Fe, Co, Ni, Cu, Zn, As, Sr, Mo, Cd, Ba, and Pb), the sediment samples were digested as follows: 100 mg of the sediment sample was digested by 1 mL of 50% HNO_3_ and 3 mL of HF in a tightly closed polytetrafluoroethylene vessel, and kept on a hot plate at 160°C for 48 h. After evaporation to dryness, 1 mL of 55% HClO_4_ was added to the vessel, which was then heated at 160°C until the complete evaporation of the acid was achieved. After the sample cooled to room temperature, 1.5 mL of 50% HNO_3_ was added, and the sample was heated at 160°C for 12 h and then cooled to room temperature. Afterward, the solution was diluted with 10% HNO_3_ (Zheljazkov and Nielsen 1996) and the elemental composition was determined using inductively coupled plasma mass spectrometry (ICP-MS) (PerkinElmer Optima 5300 DV device). The composition of cations and anions in the porewater was measured using ion chromatography on a Metrohm 850 (Metrohm, Switzerland). Laboratory analytical calibration verification (ICV 1640a) was performed before elemental analysis. Quality control was conducted using certified reference material (CRM) PACS-2, reagent blanks, spiked samples, collected in Esquimalt by the National Research Council Canada (NRC) for every 10 samples. These CRMs were verified within 3% of their expected values for all elements.

#### Statistical analysis

IBM SPSS Statistics, Version 28.0.1.0 (142), was used to perform one-way analysis of variance (ANOVA) and principal component analysis (PCA). All results are expressed as mean ± standard error (SE). Data were tested for normality and homogeneity of variance to ensure that they satisfied the assumptions of parametric methods. ANOVA was used to assess differences among the sites in terms of sediment characteristics.

## Results

### Porewater analysis of total organic carbon, major ions, and trace elements

**Fig. 3:**
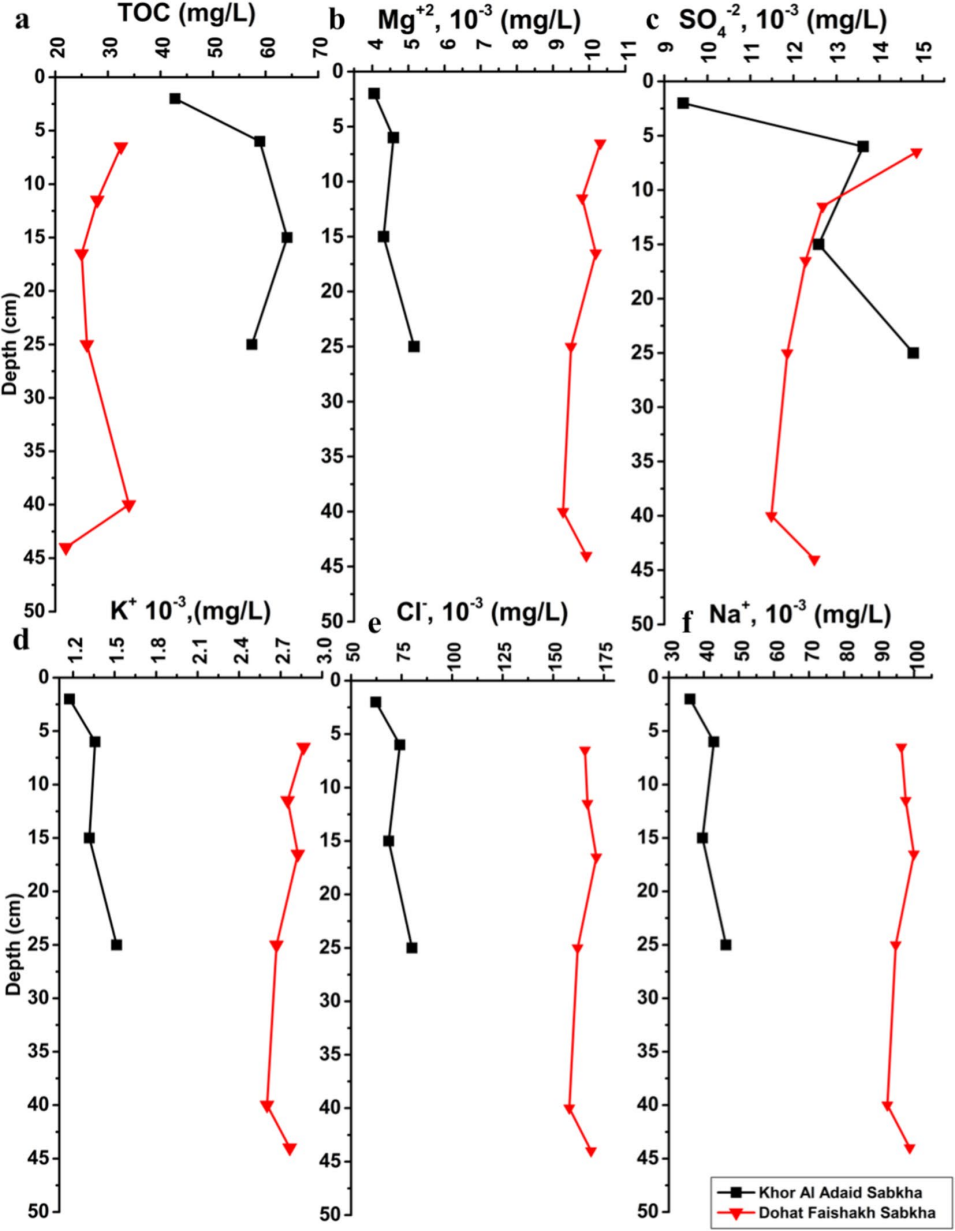
Depth profiles of TOC and dissolved ions in porewater in the Khor Al-Adaid sabkha and the Dohat Faishakh sabkha.** a** TOC (mg/l),** b** Mg+2,** c** SO4-2,** d** K+,** e** Cl-,** f** Na+

Total organic carbon (TOC) values recorded in porewater at the Khor Al-Adaid sabkha (42.8–64.1 mg/L) were higher than those found in the Dohat Faishakh sabkha (22–34 mg/L) (Fig. 3). TOC increased with depth in Khor Al-Adaid and has two minima at the depths of 17 and 45 cm in Dohat Faishakh. The concentrations of cations Na^+^, K^+^, Mg^+2^, and anion Cl^−^ in Dohat Faishakh were higher than those in Khor Al-Adaid (Fig. 3). Nitrate (NO_3_^−^) concentrations ranged (0–2.33 ⋅10^–3^ mg/L) in Khor Al-Adaid, while it was below the detection limit in Dohat Faishakh (Table S2). Sulfate (SO_4_^−2^) concentrations exhibited variability in Khor Al-Adaid (~ 9–15⋅10^–3^ mg/L) and Dohat Faishakh (~ 11–15⋅10^–3^ mg/L), declining with depth in the Dohat Faishakh sabkha and, in contrast, increasing with depth in the Khor Al-Adaid sabkha. Fe and Mn, as well as Cr, Cu, V, and Ni, are found in higher concentrations in the Dohat Faishakh sabkha.

### Mineralogical composition

**Fig. 4:**
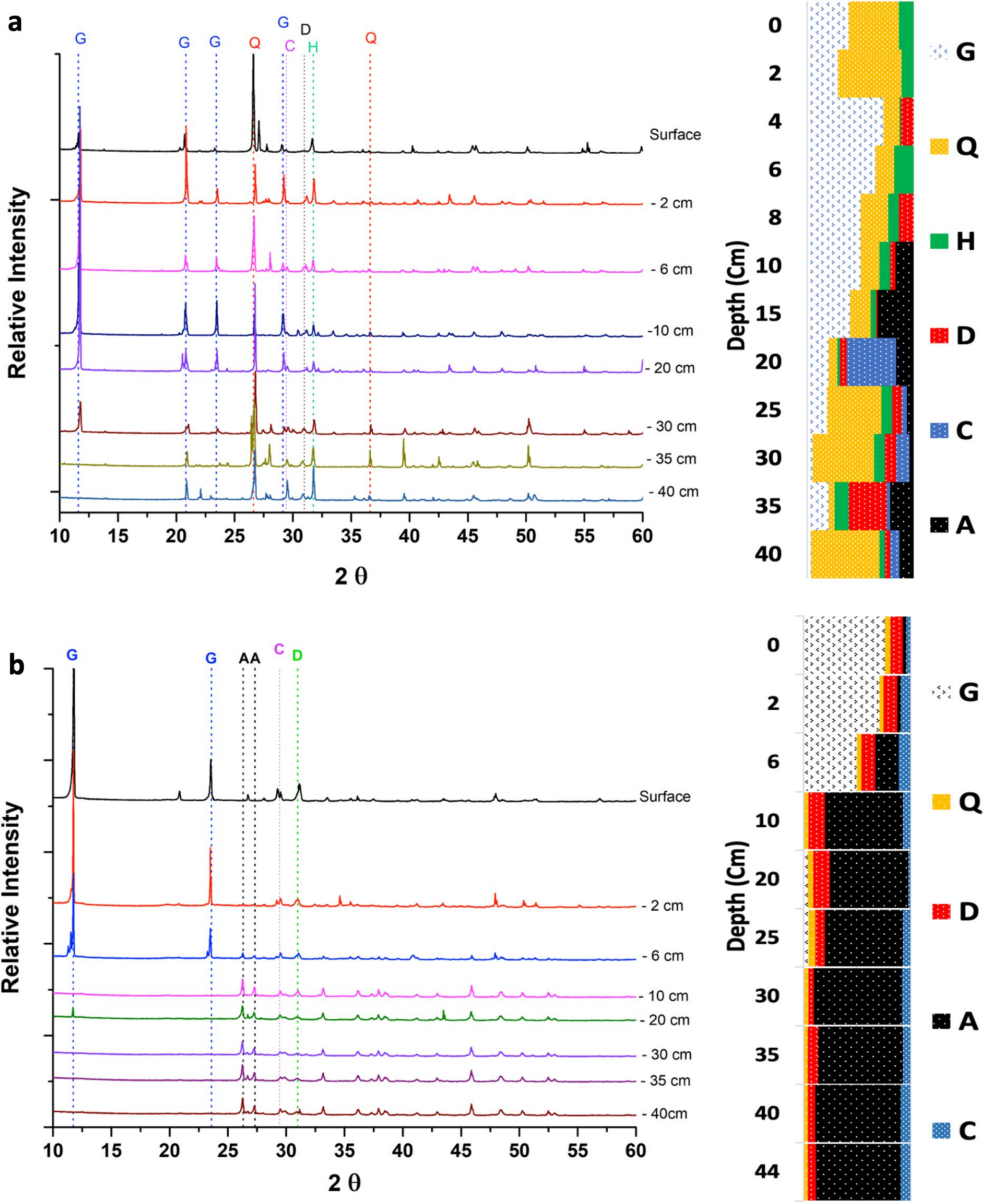
X-ray diffractograms and mineralogy patterns based on the semi-quantitative calculated for the **a** Khor Al-Adaid sabkha and **b** Dohat Faishakh sabkha. *Q* Quartz, *C* Calcite, *D* Dolomite, *A* Aragonite, *G* Gypsum, *H* Halite

The results of the XRD analysis (Fig. 4) indicate the abundance of gypsum and quartz minerals in all layers of the Khor Al-Adaid sediments until 40 cm. In contrast, the Dohat Faishakh sediments were characterized by aragonite-dominated layers; the aragonite was present in deeper layers of the sediment core, starting at a depth of 10 cm and continuing down to the bottom layers until 44 cm. Dolomite was present in the Khor Al-Adaid sabkha at 4 cm and 8 cm, which is in contrast to the Dohat Faishakh sabkha, where dolomite is shown to be present in all layers. In Khor Al-Adaid, calcite was abundant at 20 cm and was present at 30 cm and 40 cm; in Dohat Faishakh, calcite was shown to be distributed in all layers.

### Total organic and inorganic carbon in sediments

**Fig. 5:**
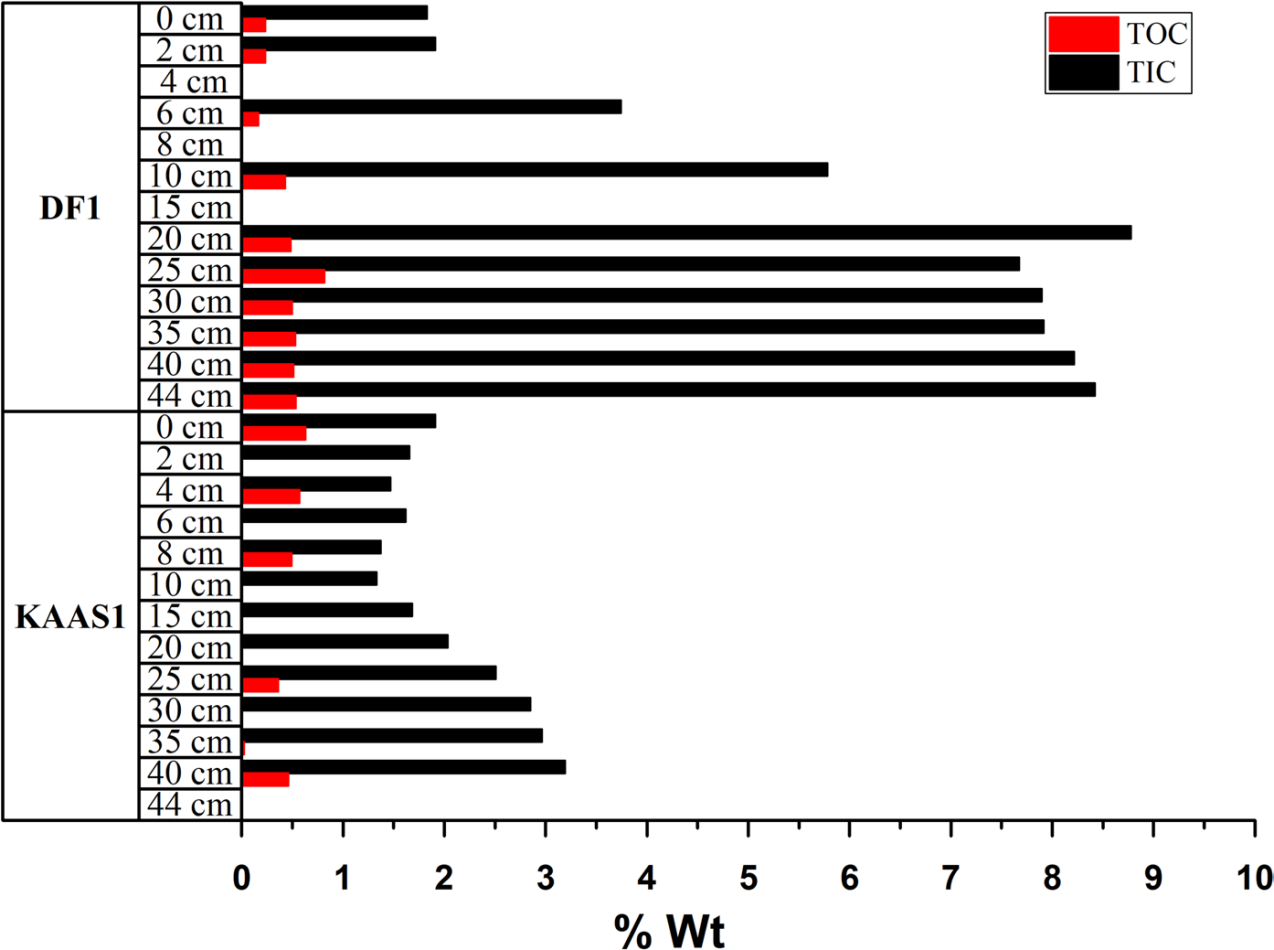
Depth profiles of proportions of TOC (%) and TIC (%) with respect to TC in the **a** Khor Al-Adaid sabkha and **b** Dohat Faishakh sabkha

**Fig. 6:**
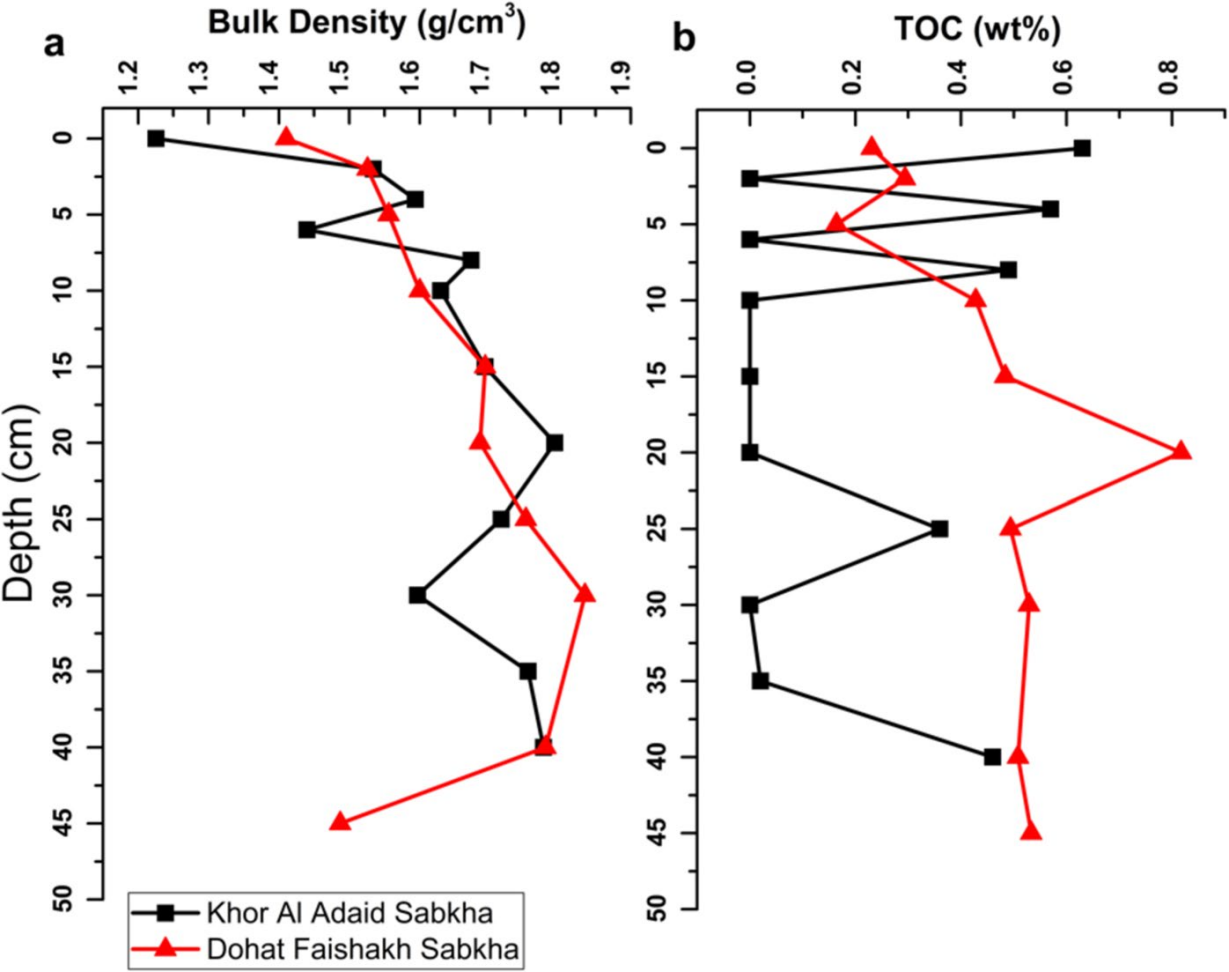
Depth profiles of bulk density (**a**) and TOC (wt%) in sediment samples collected from the Khor Al-Adaid sabkha and Dohat Faishakh sabkha

The total inorganic carbon (TIC) in the Dohat Faishakh sediment is higher overall (1.83–8.77 wt%) than that in the Khor Al-Adaid sediment (1.33–3.19) (Fig. 4). Statistical analysis (Table 2) confirms that the TIC in both sabkhas increased significantly with depth (0.904, P value < 0.001; and 0.875, P value < 0.001) for Khor Al-Adaid and Dohat Faishakh, respectively. There was no significant difference between the bulk density of the sediments for the two sabkhas (Table 3, Fig. 6). In Khor Al-Adaid, TOC varied between 0.6 and 0 wt%, whereas in Dohat Faishakh, TOC increases steadily with depth, reaching the highest concentration at 20 cm (0.8 wt%) (Fig. 5). The TOC ranged from 0.16 to 0.82 wt% in the Dohat Faishakh sediments, while the TOC reached a maximum of 0.63 wt% in Khor Al-Adaid (Fig. 6).

**Table 2:** Analysis of variance TIC (%), TOC (%), and mineral composition in the Khor Al-Adaid and Dohat Faishakh sabkhas

			Depth (cm)	Gypsum	Quartz	Goethite	Dolomite	Calcite	Aragonite
Khor Al-Adaid	TICwt%	Correlation	0.904^**^	− 0.823^**^	0.438	− 0.193	0.426	0.247	0.199
		P value	< 0.001	0.001	0.154	0.548	0.167	0.438	0.536
	TOCwt%	Correlation	− 0.172	0.259	0.247	0.247	− 0.031	− 0.256	− 0.464
		P value	0.592	0.417	0.439	0.439	0.923	− 0.153	0.128
Dohat Faishakh	TIC wt%	Correlation	0.875^**^	− 0.954^**^	0.159	0.358	− 0.410	0.674	0.958^**^
		P value	< 0.001	< 0.001	0.661	0.310	0.239	− 0.198	< 0.001
	TOCwt%	Correlation	0.672^*^	− 0.750^*^	0.511	0.248	− 0.432	0.584	0.762^*^
		P value	0.033	0.012	0.131	0.490	0.212	− 0.153	0.010

^*^Correlation is significant at the 0.05 level (2-tailed)

^**^Correlation is significant at the 0.01 level (2-tailed)

The bold numbers indicate a significant correlation

**Table 3:** Bulk density and C_org_ stock in the top 44 cm(^a^) and in 1 m depths(^b^) of the sediments collected from the Dohat Faishakh and Khor Al-Adaid sabkhas

Study site	Bulk density (g cm^−3^)	C_org_ stock^a^ (Mg C ha^−1^)	C_org_ stock^b^ (Mg C ha^−1^)*
Khor Al-Adaid	1.62 ± 0.04	13.75 ± 0.38	31.25 ± 0.86
Dohat Faishakh	1.63 ± 0.04	37.17 ± 0.81	84.48 ± 1.84
P value	0.875	0.004	0.004

Values are mean ± SE

^*^Projected C_org_ stocks were extrapolated to top 1 m sediment depth

### Organic carbon and inorganic carbon stock

C_org_ stock has been estimated at 13.75 ± 0.38 Mg C ha^−1^ in the Khor Al-Adaid sabkha and significantly higher (37.17 ± 0.81 Mg C ha^−1^) in the Dohat Faishakh sabkha for 0. 44 m (Table 3). Organic carbon stock extrapolated to a sediment depth of 1 m was found to be 31.25 ± 0.86 Mg C_org_ ha^−1^ in Khor Al-Adaid and 84.48 ± 1.84 Mg C_org_ ha^−1^ in Dohat Faishakh. As expected, there was a difference in C_inorg_ stock in 1 m sediment depth between the Khor Al-Adaid (458.98 Mg C ha^−1^ in 1 m) and the Dohat Faishakh sabkhas (1191.89 Mg C ha^−1^).

**Fig. 7:**
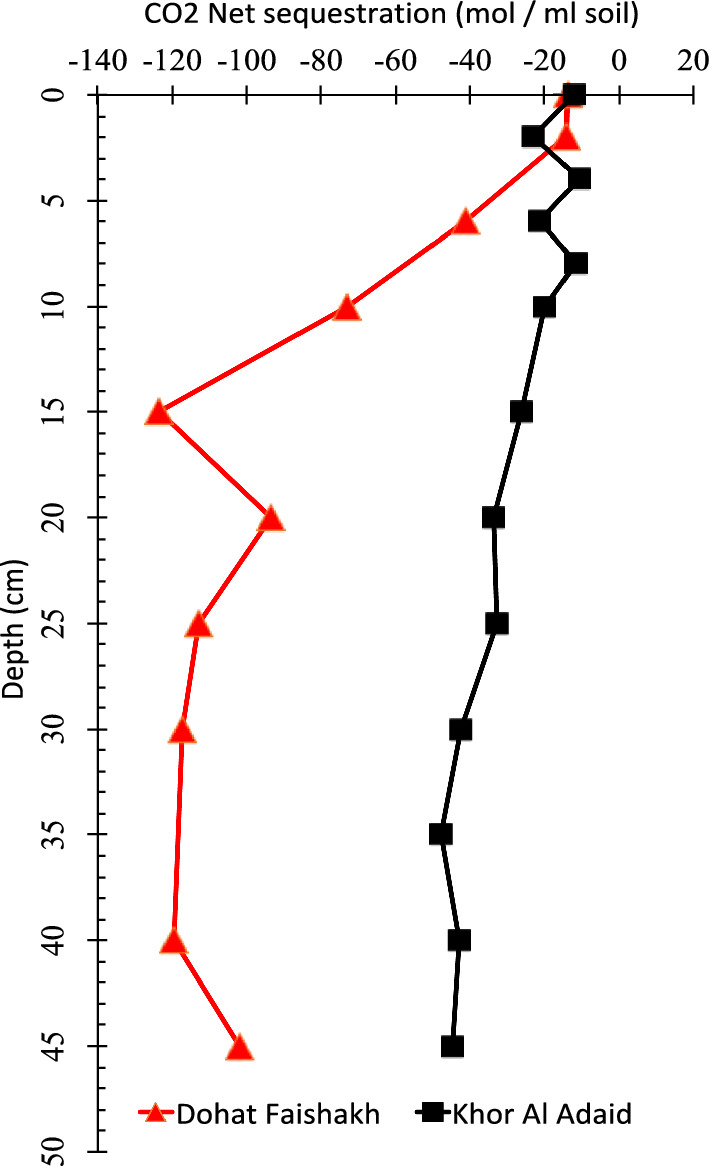
Depth profile of CO_2_ net sequestration (mol mL soil^−1^) in the Khor Al-Adaid and Dohat Faishakh sabkhas

CO_2_ levels measured in the two sabkhas showed that CO_2_% is higher in deeper sediments than in upper layers (Table 4). In the Dohat Faishakh sabkha, CO_2_% was 0.03% at 5 cm and 0.06% at 22 cm. In the Khor Al-Adaid sabkha, CO_2_% was 0.02% at 5 cm and 0.05% at 22 cm. The calculated values of CO_2_ sequestration showed negative values of CO2, indicating CO2 evasion for both sabkhas. It appears that CO₂ is released across all layers (Fig. 7), with CO₂ net sequestration being lower in the Khor Al Adaid Sabkha. In contrast, the Dohat Faishakh Sabkha exhibited greater CO₂ release, with the highest evasion occurring at a depth of 15 cm, calculated as − 140 mol mL⁻^1^ of soil.

**Table 4:** Gas samples collected from different depths of the two sabkhas and analyzed for isotopic composition and CO_2_

Site name	Depth	Latitude	Longitude	Geochemical background	CO_2_%
Dohat Faishakh	5 cm	50.96463	25.638983	Carbonate	0.03
Dohat Faishakh	22 cm	50.96463	25.638983	Carbonate	0.06
Khor Al-Adaid	5 cm	51.326625	24.646092	Siliciclastic	0.02
Khor Al-Adaid	20 cm	51.326625	24.646092	Siliciclastic	0.05

## Discussion

### Carbon retention in carbonate and siliciclastic environments

By storing substantial amounts of organic carbon in coastal sediments for long periods, BCEs play a crucial role in sequestering carbon dioxide and mitigating climate change (Macreadie et al. 2019). One of the systems that have recently been studied for their role in long-term storage of organic carbon are the mudflats (sabkhas) (Eid et al. 2022, 2023; Al disi et al. 2023). Mudflats (sabkhas) are distributed all around the world, with predominance in the Arabian Gulf and Red Sea regions (Table 5). In this study, organic carbon was found to vary between two Arabian Gulf sabkhas with contrasting geological backgrounds: siliciclastic (Khor Al-Adaid) and carbonate (Dohat Faishakh). Organic carbon was found to be highest at 0.63% wt% in Khor Al-Adaid and 0.82 wt% in Dohat Faishakh, which is consistent with what has previously been reported in the sabkhas (Brauchli et al. 2016; Al disi et al. 2023). In the two studied sabkhas, soil organic carbon stock accounted for 31.25 ± 0.86 Mg C_org_ ha^−1^ in Khor Al-Adaid and 84.48 ± 1.84 Mg C_org_ ha^−1^ in Dohat Faishakh. The observed carbon stocks fall within the range of other carbon stocks reported from sabkhas located in the United Arab Emirates (51–120.5 Mg C ha^−1^) (Schile et al. 2017; Eid et al. 2022). However, these numbers are lower than what observed by Al disi et al. 2023), which reported 67.8 ± 18.1 Mg C ha^−1^ for the Khor Al-Adaid sabkha and 109.1 ± 7.1 Mg C ha^−1^ for the Dohat Faishakh sabkha, the difference in numbers might be attributed to heterogeneity in space and time, since the sample sites in two studies differs as well as sampling times. These variations in carbon stocks suggest that soil organic carbon content is heterogeneously distributed. Carbon stocks in other coastal ecosystems, such as salt marshes, are higher, ranging from 233.86 to 624.13 Mg C m^−2^ (Kim et al. 2022). For other ecosystems within the Arabian Gulf, reported carbon stocks along Saudi Arabian coastal areas range between 62 and 127 Mg C ha^−1^ in seagrasses, between 51 and 113 Mg C ha^−1^ in mangroves, and between 53 and 113 Mg C ha^−1^ in salt marshes (Cusack et al. 2018).

**Table 5:** Overview of sabkha areas around the world

Sabkha name	Geological background	Area (km^2^)	Location	References
Dukhan Sabkha	Carbonate	73	Qatar	Aref et al. (2013), Rivers et al. (2020)
Um-Said Sabkha	Gypsum and Dolomite	350	Qatar	Aref et al. (2013), Rivers et al. (2020)
Dohat Faishakh Sabkha	Carbonate	20	Qatar	Aref et al. (2013), Rivers et al. (2020)
Khor Al-Adaid Sabkha	Siliciclastic	705	Qatar	Aref et al. (2013)
Abu Dhabi Sabkha	Carbonate	1654	United Arab Emirates	Warren (2016)
Al Zeeb Sabkha	Siliciclastic	10	Saudi Arabia	Aref et al. (2013)
Ras-Shukheir Sabkha	Gypsum	15	Egypt	Warren (2016)
El Haman Sabkha	–	79.3	Egypt	Warren (2016)
El, Qaa Sabkha	–	4.9	Gulf of Suez, Egypt	Embabi (2018)
Bardawil Lagoon Sabkha	Siliciclastic	135.9	Egypt	Embabi (2018)
Al Khawwari Coast Sabkha	–	5	Saudi Arabia	Al-Hurban and El-Gamily (2013)
NW coast	–	5	Australia	Warren (2016)
Tickera region	–	1.3	South Australia	Warren (2016)
Guerrero Negro	–	373	Mexico	Warren (2016)
Laguna Madre mudflat	Carbonate	106.3	Texas, USA	Warren (2016)
Ras Shoukair	Gypsum	15	Egypt	Aref et al. (2013)
Cyanobacterial Mats of the Exmouth Gulf	–		Western Australia	Hickey et al. (2023)

Carbonate-dominated seagrass ecosystems are consistently rich in organic carbon. Miyajima et al. (2015) found that carbonate-dominated seagrass meadows were richer in organic carbon (ca. 1200 μmol C g^−1^) compared to meadows dominated by silicate sand and aluminosilicate mud (< 630 μmol C g^−1^), although organic carbon in seagrass meadows is primilary affected by mangrove-derived organic carbon, however, this finding suggests that the minerology of the sediments, composed of calcium carbonates are more favorable for organic carbon accumulation due to the porus nature of carbonate sediments that enhance carbon preservation and slower decomposition rates, which can be applied to coastal sabkhas as well. The presence of Mg^2+^ and Ca^2+^ contributes to the formation of aggregates, resulting in lower rates of microbial oxidation of organic carbon (Deb and Mendal 2021). Long-term storage of organic carbon has been found to be affected by several environmental factors, such as the mud content of the sediments and hydrodynamic forces (Kim et al. 2022; Lavery et al. 2013; Mazarrasa et al. 2018; Samper-Villarreal et al. 2016).

Studies on blue carbon in the Arabian Gulf lack data on CO_2_ evasion and carbon dating, which are crucial for the identification of BCEs. In this study, organic carbon from Khor Al-Adaid cores date to 7150 BP at 40 cm, which is consistent with what has been reported in previous studies (Engel et al. 2014; Kassler 1973; Lambeck 1996; Lokier 2012; Rivers et al. 2020). The Dohat Faishakh sabkha, on the other hand, is an evaporitic environment consisting mainly of carbonate minerals that formed during the Holocene (4000–6000 years ago) (Brauchli et al. 2016). Sediments were dated to 4839 ± 79 years BP at 29 cm (Rouwendaal 2017). Gas composition from the sabkhas showed more CO_2_ at 20 cm sediment depths than at the surface sediment. In the Khor Al-Adaid sabkha 0.05% CO_2_ were found at 20 cm and 0.02%—at 5 cm; and in the Dohat Faishakh sabkha the analysis showed 0.06% CO_2_ at 20 cm and 0.03% at 5 cm. Higher percentages of CO_2_ in deeper sediments can be linked to organic matter decomposition occurring through anaerobic processes (Brooker et al. 2014).The important criterium for an ecosystem to be associated with BCE is the richness of sediments with organic carbon and the capacity to retain it over long periods. Organic carbon content and retention is related to the geochemical conditions and geological background of the sediments (Miyajima et al. 2015). The mineralogy of Khor Al-Adaid shows that this area is dominated by siliciclastic sand, mostly comprised of quartz and gypsum, but it also includes some carbonate minerals such as dolomite and aragonite, as well as the evaporites gypsum and halite (DiLoreto et al. 2019; Rivers et al. 2020). Compared to carbonate-dominated sabkhas, siliciclastic carbonate sabkhas have a more diversified range of mineral types (Fig. 4); this can be linked to an uneven distribution of organic matter the Khor Al-Adaid in comparison to the Dohat Faishakh (Fig. 6). Indeed, in soils and sediments, OC is often associated with minerals, consequently, the longterm stability of organic carbon in soils depends on minerals-organic matter interactions, triggered by such processes as sorption, ligand and iron exchange, aggregation. Thus, the presence of minerals-organic matter aggregates can inhibit organic matter decomposition while minerals can serve as a protective layer against microbial attack. In coastal dynamic areas, such as sabkhas, the redox conditions, salinity and input of allochthonous materials, impact minerals-OM interactions depending on minerals stability in respect of redox conditions and salinity (Dong, et al. 2022). Thus, geological background which defines mineral- organic matter interactions can impact OC retention.

### Mechanism of organic carbon preservation in sabkhas

Understanding the dynamics of organic carbon in sabkhas is essential for the assessment of blue carbon. Based on measurements of C_org_ stocks within the sabkha, it was observed that these environments are well within the range of currently established BCEs (Table 2) and therefore will have a significant impact on carbon dynamics. Interestingly, the two studied sabkhas are colonized with microbial mats rather than vegetation, which is observed in traditional BCE environments (Mcleod, et al. 2011; Lovelock & Duarte 2019). Thus, the sabkha environments from this study represent a unique set of mechanisms for organic carbon preservation. Intertidal microbial mats have previously been identified as a potential blue carbon ecosystem, which can store carbon in amounts comparable to that of vegetated blue carbon ecosystems (Schile et al. 2017). Salinity in the Dohat Faishakh sabkha reaches above 300‰, while salinity values in the Khor Al-Adaid sabkha range between 48‰ and 140‰ during the year (DiLoreto et al. 2019, 2021; Rivers et al. 2020). High salt stress has been shown to stimulate the production of extracellular polymeric substances (EPS) by bacteria (Chambi et al. 2021; Seesuriyachan et al. 2012). EPS have been found to have a positive correlation with organic carbon preservation and play a significant role in blue carbon sequestration (Liu et al. 2022). The functional groups within EPS, such as carboxyl, hydroxyl, and amino groups, act as nucleation sites for mineral formation, particularly carbonates (Liu et al. 2020; Zhu and Dittrich 2016). EPS play a dual role in ion binding and precipitation by exhibiting a high affinity for essential metal cations like calcium and magnesium. Binding of ions on EPS leads to supersaturation in the surrounding environment, promoting the nucleation and growth of carbonate crystals (Paulo et al. 2020). Microbial activity within EPS matrices influences local geochemical conditions through metabolic byproducts, altering pH, and carbonate ion concentration (Robles-Fernández et al. 2022), EPS also act as a microbial glue, binding cells together to form biofilms that trap and concentrate minerals (Hilmi et al. 2021). Taking into account inorganic carbonate, the calculated CO_2_ net sequestration indicated that both sabkhas are evading CO_2_ into the atmosphere. Thus, carbonate formation is negated C_org_ accumulation in carbonaceous sabkha and offset CO2 sequestration.

**Fig. 8:**
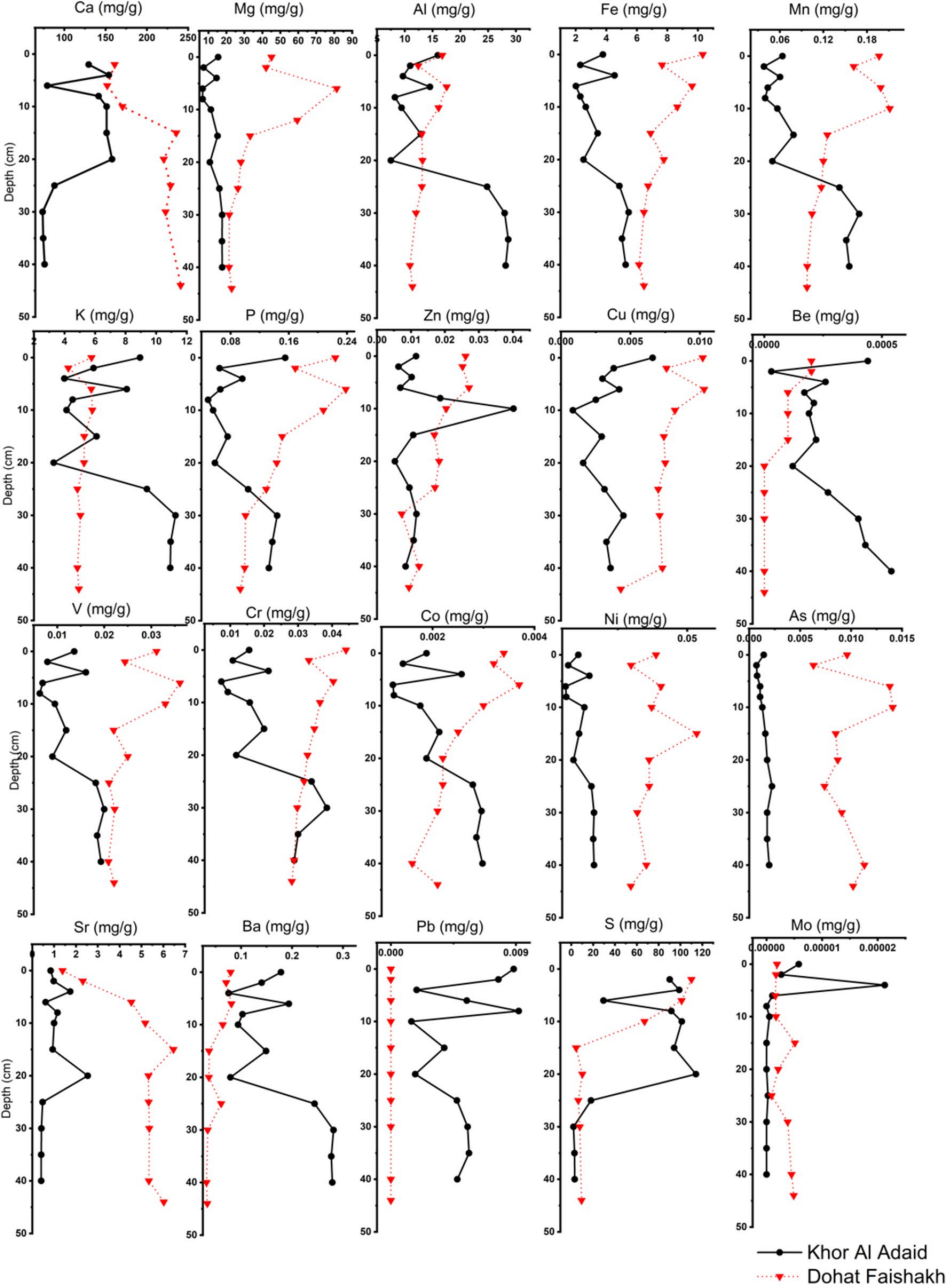
Depth profiles for major (Ca, Mg, S, and Fe) and trace (Mn, P, Sr, and Mo) elements found in sediments sampled from the Khor Al-Adaid and Dohat Faishakh sabkhas

The combination of processes promoting mineralization in the presence of microbial mats can lead to organic matter preservation as well. Studies on the Khor Al-Adaid sabkha have found small crystals of dolomite (CaMg(CO₃)₂) clusters within cyanobacterial mats (Paulo and Dittrich 2013). Dolomite has also been reported within the microbial mats of the Dohat Faishakh sabkha, suggesting that its formation is a result of microbially induced mineralization (Brauchli et al. 2016). The presence of dolomite and magnesium carbonate minerals precipitated in microbial mats can lead to the preservation of organic molecules in sediments. The natural topsoil properties greatly impact the organic carbon content in sediment, since the vegetation and microbial mats at the topsoil govern organic carbon mineralization and can trigger inorganic carbon formation (Eid et al. 2023). Consequently, the destroy of unique features such as microbial mats and sabkhas through anthropogenic activities, for example driving in sabkhas, impact carbon preservation, leading to the release of carbon dioxide and methane into the atmosphere, sabkhas have been suggested as the locations with highest methane concentrations in coastal sites of Southeastern Arabian Peninsula based on satellite-derived data (Francis et al. 2023). Furthermore, modelling approach for mudflats and salt flats on India showed that their methane emission of 0.08Tg is ca. 80% of the total anthropogenic emissions for 2000–2021 (Agarwal and Garg 2009).Redox conditions in sediments play a crucial role in the fate of organic carbon (Burdige 2007). Trace metal depth analysis (Fig. 8) of the two sabkhas provides insight into the different geochemistry of both sabkhas. The elevated amount of trace metals in solid matter in sediments is often indicative of higher redox potential, and harsher redox conditions in sediments (Reimers et al. 2013).

Organic carbon preservation is generally higher under redox and anaerobic conditions, as a larger proportion of organic matter can be preserved without complete mineralization (Burdige 2007). Elemental analysis reveals that concentrations of iron and manganese are higher in the Dohat Faishakh sabkha compared to the Khor Al-Adaid sabkha. This suggests that the sediments in Dohat Faishakh are under more reducing conditions. Additionally, SO_4_ concentrations are observed to be lower in the Dohat Faishakh sabkha than in the Khor Al-Adaid sabkha; this difference could be the result of sulfate consumption as an electron acceptor through microbial activities, which also indicates that Dohat Faishakh is under more reducing conditions. This could explain why the Dohat Faishakh sabkha has higher organic carbon content and carbon retention, as organic carbon preservation is generally higher under anaerobic conditions.

### Sabkhas in Qatar and the Arabian Gulf and challenges affecting organic carbon storage

Coastal sabkhas extend along the shoreline of Qatar with around 590 km^2^ of coastal areas (Ashour 2013). One of these sabkhas that can contribute to the organic carbon reservoir is the Dukhan sabkha, which occupies 73 km^2^, with geochemistry similar to that of the Dohat Faishakh sabkha, consisting of gypsum and dolomite (Edwards et al. 2010). Another sabkha that might also act as a carbon reservoir is Um-Said, which lies along the southeastern coast and occupies 350 km^2^; this sabkha has also been identified as a carbonate sabkha (Al-Youssef 2014). Assuming that Um-Said and Dukhan may sequester organic carbon in amounts similar to that of Dohat Faishakh, the coastal sabkhas of Qatar may attribute 3.5 Tg C to the global organic carbon budget.

Sabkha ecosystems extend along the shores of the Arabian Gulf and the Red Sea and occupy 2775 km^2^ (Loughland et al. 2018). The Abu Dhabi sabkha is one of the most studied sabkhas, extends 300 km along the shore; its sedimentology is characterized by gypsum and anhydrite in a carbonate-dominated sediment covered with microbial mats (Bontognali et al. 2010; Sadooni et al. 2010). Other sabkhas have been studied in respect to microbial and sedimentary structures along the Red Sea in both Egypt and Saudi Arabia; for example, the Al Zeeb sabkha, located in Saudi Arabia, hosts aeolian siliciclastic sands set in carbonate sediment, with more gypsum and carbonate moving toward the sea; and the Ras Shukheir sabkha, located in Egypt, is rich with microbial mats and gypsum (Aref et al. 2013). These sabkhas have a similar geochemical background to the Dohat Faishakh sabkha. Assuming they have the same organic carbon retention as Dohat Faishakh, sabkhas along the Arabian Gulf and Red Sea might contribute 23.3 Tg C to the global carbon budget. The sabkhas ecosystems is a part of tidal flats areas, which covering a small part of the global marine bottom area, ca. 0.04% (Atwood et al. 2020), carbon storage in tidal flats has been estimated as 1.1 Pg C (Chen and Lee 2022). Sabkhas of Arabian Gulf, thus can contribute ca. 2% to carbon storage, estimated with uncertainties (Al disi et al. 2023). Coastal ecosystems with vegetation cover (e.g., mangroves, seagrass meadows and salt marshes) are better studied and their role as important C reservoirs is well established, but similar assessments of tidal mudflats, vulnerable ecosystems under the threats of anthropogenic pressure, are missing (Chen and Lee 2022).The Arabian Gulf is surrounded by important coastal ecosystems such as salt marshes, mangroves, mudflats, and sabkhas, which are covered with productive microbial mats (Burt 2014). Thus, the coastal ecosystems around the Arabian Gulf may contribute largely to the blue carbon pool; however, urbanization and coastal development pose significant threats to the ecosystems and organic carbon retention. Two-thirds of Arabian Gulf sabkhas have been lost to urban development, resulting in the Arabian Gulf being considered one of the most degraded marine ecosystems (Burt 2014). The preservation and restoration of sabkhas and other intertidal ecosystems is important for increasing carbon sequestration. Efforts from policy makers have been directed to preserve these ecosystems, since recognition of the importance of mangroves, salt marches and seagrass (Nellemann et al. 2009). This study shows that sabkhas have the potential to contribute to organic carbon preservation and are required proper management and restoration planning.

## Conclusion

This study shows variability in organic carbon storage between two sabkhas (mudflats) with contrasting geochemical backgrounds: the Khor Al-Adaid sabkha and the Dohat Faishakh sabkha. The study reveals variations in organic carbon content between the sabkhas, with Khor Al-Adaid having reported carbon stocks of 31.25 ± 0.86 Mg C_org_ ha^−1^, and carbon stocks of 84.48 ± 1.84 Mg C_org_ ha^−1^ having been observed within the Dohat Faishakh sabkha. Compared to other coastal ecosystems globally, carbon stocks in the Arabian Gulf sabkhas are relatively lower, indicating potential differences in carbon sequestration capacities.

With the lack of significant vegetation in sabkhas, carbon cycling relies primarily on microbial activity; hence, the differences in carbon sequestration can be attributed to several environmental factors, such as sediment composition, mud content, and hydrodynamic forces. Additionally, redox conditions in sediments, reflected in trace metal depth analysis, indicate higher iron and manganese concentrations in the Dohat Faishakh sabkha, suggesting more anaerobic conditions. This aligns with the higher organic carbon content and retention observed in Dohat Faishakh, as anaerobic conditions generally favor organic carbon preservation.

Our findings reveal that coastal sabkhas have had organic carbon preserved in their sediments for thousands of years and had potential to retain organic carbon. However, CO_2_ evasion calculations show that both sabkhas are evading CO_2_, acting as net releasers, stressing a critical role of inorganic carbon estimation for carbon sequestration. Consequently, geological background together with the natural topsoil coverage may be key players in carbon budget. Losing natural vegetation coverage including microbial mats have consequences for carbon sequestration. The sabkhas coastal ecosystems are disappearing giving space to urban development. Even though detailed impact of sabkhas on a global carbon budget requires further investigation, the protection of the sabkha environment is critical to keep these unique ecosystems alive.

## Supplementary Information

Below is the link to the electronic supplementary material.

Supplementary file1 (DOCX 122 KB)

## Data Availability

The datasets generated during and/or analysed during the current study are available upon a request.
