# Supplementary material for: Do coastal salt mudflats (sabkhas) contribute to the blue carbon sequestration?
Source: Biogeochemistry. 2025 Jan 15;168(1):15. doi: 10.1007/s10533-024-01204-5 (PMC11735541; doi:10.1007/s10533-024-01204-5)
Supplement: Supplementary file 1 — Supplementary file1 (DOCX 122 KB) [file 10533_2024_1204_MOESM1_ESM.docx]

**Do coastal salt mudflats (sabkhas) contribute to the blue carbon sequestration?**

Hadil Elsayed^1^, Zulfa Ali Al Disi^1^, Khaled Naja^1^, Ivan Strakhov^2^, Scott O.C. Mundle^3^, Hamad Al Saad Al-Kuwari^1^, Fadhil Sadooni^1^, Zach Diloreto^2^, Jassim Abdulla A Al-Khayat^1^, Maria Dittrich^1, 2^

*^1^Environmental Science Center, Qatar University, P.O. Box 2713, Doha, Qatar*

*^2^Biogeochemistry Group, Department of Physical and Environmental Sciences, University of Toronto Scarborough, 1065 Military Trail, Toronto, Ontario, M1C 1A1, Canada*

*^3^University of Windsor, Ontario, Canada*

Corresponding Author Email: [m.dittrich@utoronto.ca](mailto:m.dittrich@utoronto.ca)

**Supplementary materials**

# Results

## Trace metal depth profiles in solid phase

The range and average concentrations of major and trace elements are given in Table S1 and Figure 3. The concentrations of major (Mg, Ca, S, K, Fe, and Al) and trace (As, Ba, Be, Co, Cr, Cu, Mo, Ni, P, Pb, Sr, Zn, and V) elements in Dohat Faishakh were higher than those in Khor Al-Adaid (Figure 3) at the surface sediment. Moreover, the behavior of the elements across depth varies between the two locations, exhibiting trends of metal concentrations through sediment depth (0–44 cm) (Figure 3). Most of the element concentrations started to stabilize at depths below 25 cm in both locations.

Calcium (Ca) depth profiles showed similar patterns at Khor Al Adaid as at Dohat Faishakh. The concentration of Ca ranged from 73.76–157 mg/g in Khor Al-Adaid, while it ranged from 152–240 mg/g in Dohat Faishakh. In both locations, higher concentrations were detected in the upper layers, followed by depletion with depth (Figure 3). In Khor Al-Adaid, sulfur concentrations exhibit similar behavior to Ca: sulfur concentrations reach 90.2 mg/g at 2 cm; reach their highest concentration at 114 mg/g at 20 cm; and decrease with depth, reaching 2.04 mg/g at 30 cm and 3.1 mg/g at 40 cm. Magnesium (Mg) concentrations showed significant differences between the two sabkhas. In Dohat Faishakh sabkha, low Mg concentrations were found at the surface layer, reaching a maximum at 7 cm and at the deeper sediment, while in Khor Al-Adaid, Mg concentrations fluctuated and stabilized with depth. Aluminum (Al) concentrations fluctuated between 10.9–17.5 mg/g and between 20–25 mg/g in Dohat Faishakh and Khor Al-Adaid, respectively. Manganese (Mn) and iron (Fe) followed a similar trend in the Khor Al-Adaid sabkha, increasing with depth. In the Doha Faishakh sabkha, both metal concentrations decreased with depth. Molybdenum (Mo) concentrations ranged between 0 and 0.002 mg/g in Khor Al-Adaid, with one peak at the depth of 5 cm, then Mo concentrations expressed little fluctuation with increased depth in the Khor Al-Adaid sediments. Higher Phosphorus (P) concentrations between 0.16–0.24 mg/g were found in the surface layers of Khor Al-Adaid and Dohat Faishakh; P dropped at the depth of 2 cm to 0.06 and 0.16 mg/g, respectively. However, in Dohat Faishakh, concentrations of P increased to 0.23 mg/g at the depth of 6 cm and dropped gradually to the lowest concentration of 0.09 mg/g at the depth of 44 cm. In Khor Al-Adaid, P concentration increased at the depth of 30 cm and reached 0.14 mg/g at the depth of 40 cm.

## Radiocarbon analysis

The dating of carbonate samples from 35 and 40 cm from the Khor Al-Adaid sabkha (Table S3) indicated that carbonates formed in 35 cm are 22100 BP, while carbonates from 40 cm date to 17400 BP. Δ^14^ C represents the difference in C^14^, from modern C^14^ levels, indicating that carbonates at 35 cm are slightly more depleted than samples at the depth of 40 cm.

## Principal component analysis (PCA) for element concentrations, total inorganic carbon (TIC), and total organic carbon (TOC)

Significant close relationships were found among the bulk density, TOC, and strontium, molybdenum, and calcium concentrations in the Dohat Faishakh sediments; however, TOC appears to have no relationship with other elements in the Khor Al-Adaid sediments (Figures S2a, b). PCA of the element concentrations in the porewater indicate a negative relationship between TOC and depth in the Dohat Faishakh sabkha (Figure S1d). In comparison, PCA showed no relationship between TOC and depth, neither in the porewater (Figure S1d) nor in the solid contents (Figures S1a, c; Table 3) of the Khor Al-Adaid sabkha.

**Figure S1.** Biplots of principal component analyses for constituents: (a) solid phase contents from Khor Al-Adaid; (b) solid phase contents from Dohat Faishakh; (c) element contents in porewater from Khor Al-Adaid; and (d) porewater contents from Dohat Faishakh sabkha.

**
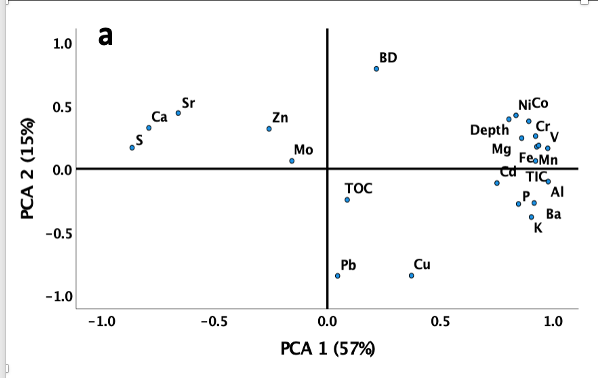
**

**
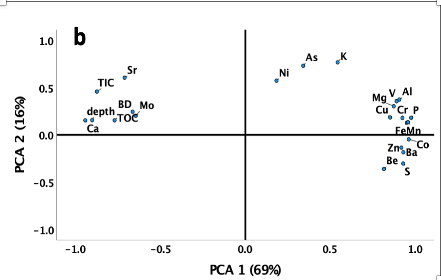
**

**
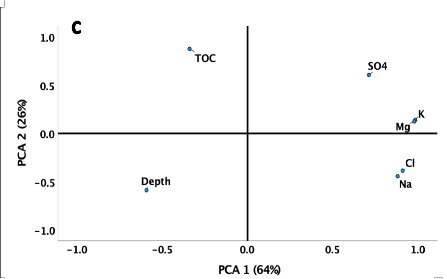
**

**
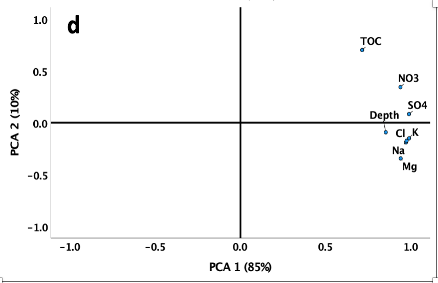
**

**Table S1.** Range and average concentrations (mg/g) of major and trace metals in sediments of Khor Al-Adaid and Dohat Faishakh sabkhas.

| Concentration (mg/g) | Khor Al-Adaid Sabkha | | Dohat Faishakh Sabkha | |
| --- | --- | --- | --- | --- |
|  | Range | Average | Range | Average |
| **Mg** | 7-18 | 13 | 22-81 | 38 |
| **Ca** | 74-158 | 117 | 152-241 | 202 |
| **Al** | 8-29 | 17 | 11-18 | 14 |
| **Fe** | 2-5 | 4 | 6-10 | 5 |
| **S** | 2-114 | 54 | 4-110 | 44 |
| **K** | 3-11 | 7 | 4-6 | 5 |
| **Sr** | 0.4-2 | 1 | 1-6 | 5 |
| **Mn, 10^-3^** | 38-170 | 87 | 101-210 | 143 |
| **P, 10^-3^** | 50-156 | 95 | 90-240 | 155 |
| **Ba, 10^-3^** | 79-281 | 175 | 32-80 | 53 |
| **Zn, 10^-3^** | 5-40 | 12 | 7-20 | 18 |
| **Cu, 10^-3^** | 0.85-7 | 3 | 4-10 | 8 |
| **Be, 10^-3^** | 0.3-0.5 | 0.27 | 0-0.2 | 0.07 |
| **V, 10^-3^** | 6-20 | 13 | 22-36 | 26 |
| **Cr, 10^-3^** | 7-39 | 20 | 28-44 | 34 |
| **Co, 10^-3^** | 1-3 | 2 | 1-4 | 3 |
| **Ni, 10^-3^** | 6-17 | 12 | 3-54 | 40 |
| **As, 10^-3^** | 0.7-2 | 1 | 6-14 | 10 |
| **Pb, 10^-3^** | 2-10 | 5 | 0 | 0 |
| **Mo, 10^-3^** | 0-20 | 3 | 1-5 | 3 |

**Table S2.** Range and average concentrations (mg/L) of major and trace metals in porewater collected from Khor Al-Adaid and Dohat Faishakh sabkhas.

| Concentration (mg/L) | Khor Al-Adaid porewater | | Dohat Faishakh Sabkha porewater | |
| --- | --- | --- | --- | --- |
|  | Range | Average | Range | Average |
| **Na^+^, 10^-3^** | 36-46 | 41.2 | 92-100 | 97 |
| **K^+^, 10^-3^** | 1.2-1.5 | 1.34 | 2.6-2.9 | 2.7 |
| **Mg^+2^, 10^-3^** | 4-5.1 | 4.5 | 9.2-10.3 | 10 |
| **Cl^-^, 10^-3^** | 62-80 | 71 | 158-171 | 165 |
| **NO_3_^-^, 10^-3^** | 0-2.33 | 1.6 | 0 | 0 |
| **SO4^-2^, 10^-3^** | 9-15 | 13 | 11-15 | 13 |

**Table S3.** Measured radiocarbon values at Khor Al-Adaid sabkha at 35 and 40 cm depths.

| **Site** | **Depth (cm)** | **Sample Type** | **D^14^ C**  **(Standard deviation)** | **Age (BP)** | **Δ^14^ C** |
| --- | --- | --- | --- | --- | --- |
| Khor Al-Adaid | 35 | Carbonate | -936.15 (-0.56) | 22100 ± 70 | -936.70 (-0.56) |
|  | 40 | Carbonate | -885.37 (-0.71) | 17400 ± 50 | -886.37 (-0.71) |
|  | 40 | Organic carbon | -607.38 (-1.48) | 7150 ± 30 | -610.78 (-1.47) |
